# Behavioural Fever Promotes an Inflammatory Reflex Circuit in Ectotherms

**DOI:** 10.3390/ijms22168860

**Published:** 2021-08-17

**Authors:** Nataly Sanhueza, Ricardo Fuentes, Andrea Aguilar, Beatriz Carnicero, Karina Vega, David Muñoz, David Contreras, Nataly Moreno, Eduardo Troncoso, Luis Mercado, Byron Morales-Lange, Sebastian Boltana

**Affiliations:** 1 Centro de Biotecnología, Departamento de Oceanografía, Universidad de Concepción, Concepción 4030000, Chile; natalysanhueza@udec.cl (N.S.); aguilar.espinoza@gmail.com (A.A.); b.carniceroarnanz@gmail.com (B.C.); karinavegadrake@gmail.com (K.V.); bio.dnmunoz10@gmail.com (D.M.); 2 Departamento de Biología Celular, Facultad de Ciencias Biológicas, Universidad de Concepción, Concepción 4030000, Chile; ricfuentes@udec.cl; 3 Biotechnology Center, Renewable Resources Laboratory, University Campus, Universidad de Concepción, Concepción 4030000, Chile; dcontrer@udec.cl (D.C.); natalymoreno@udec.cl (N.M.); etroncoso@udec.cl (E.T.); 4 Grupo de Marcadores Inmunológicos, Facultad de Ciencias, Instituto de Biología, Pontificia Universidad Católica de Valparaíso, Valparaíso 2340000, Chile; luis.mercado@pucv.cl (L.M.); byron.morales@pucv.cl (B.M.-L.)

**Keywords:** mobile ectotherm, inflammatory reflex, cholinergic neuron, anti-inflammatory, behavioural fever

## Abstract

Background: The communication between the brain and the immune system is a cornerstone in animal physiology. This interaction is mediated by immune factors acting in both health and pathogenesis, but it is unclear how these systems molecularly and mechanistically communicate under changing environmental conditions. Behavioural fever is a well-conserved immune response that promotes dramatic changes in gene expression patterns during ectotherms’ thermoregulatory adaptation, including those orchestrating inflammation. However, the molecular regulators activating the inflammatory reflex in ectotherms remain unidentified. Methods: We revisited behavioural fever by providing groups of fish a thermal gradient environment during infection. Our novel experimental setup created temperature ranges in which fish freely moved between different thermal gradients: (1) wide thermoregulatory range; T° = 6.4 °C; and (2) restricted thermoregulatory range; T° = 1.4 °C. The fish behaviour was investigated during 5-days post-viral infection. Blood, spleen, and brain samples were collected to determine plasmatic pro- and anti-inflammatory cytokine levels. To characterize genes’ functioning during behavioural fever, we performed a transcriptomic profiling of the fish spleen. We also measured the activity of neurotransmitters such as norepinephrine and acetylcholine in brain and peripheral tissues. Results: We describe the first set of the neural components that control inflammatory modulation during behavioural fever. We identified a neuro-immune crosstalk as a potential mechanism promoting the fine regulation of inflammation. The development of behavioural fever upon viral infection triggers a robust inflammatory response in vivo, establishing an activation threshold after infection in several organs, including the brain. Thus, temperature shifts strongly impact on neural tissue, specifically on the inflammatory reflex network activation. At the molecular level, behavioural fever causes a significant increase in cholinergic neurotransmitters and their receptors’ activity and key anti-inflammatory factors such as cytokine Il10 and Tgfβ in target tissues. Conclusion: These results reveal a cholinergic neuronal-based mechanism underlying anti-inflammatory responses under induced fever. We performed the first molecular characterization of the behavioural fever response and inflammatory reflex activation in mobile ectotherms, identifying the role of key regulators of these processes. These findings provide genetic entry points for functional studies of the neural–immune adaptation to infection and its protective relevance in ectotherm organisms.

## 1. Background

Animals have developed numerous adaptation strategies to survive in changing environmental conditions. This adaptive process allows them to generate physiological and behavioural responses upon exposure to natural factors such as temperature. Unlike endotherms, ectotherm organisms adjust their cellular and molecular metabolism to counteract temperature constraints [1,2]. This thermal-induced response developmental program requires the function of interconnected organismal systems and signalling. However, the nature of this response and how it is molecularly activated are not well understood in most of the organisms studied.

Under pathogen invasion and organ damage, the coordinated and bidirectional interaction between the nervous and immune system is regulated by the so-called inflammatory reflex [3,4,5]. During this response, immune molecules activate the afferent and efferent sensory neural arc to modulate a fine crosstalk between these systems [5,6], even under temperature changes [7,8]. Therefore, understanding the molecular program facilitating its activation is relevant to reveal thermoregulatory pathways functioning in fever.

In mammals, the inflammatory reflex is mediated by muscarinic receptors and cholinergic activity (reviewed in [5,9]), specifically, it is exerted through the function of the cholinergic receptor nicotinic alpha 7 subunit (Chrna7) and beta-2 adrenergic receptor (Adrb2) [10,11,12]. Additionally, the cholinergic pathway confers protection in peripheral organs, inflammatory conditions, and fever [13,14]. For instance, it suppresses excessive inflammation in the liver [15], heart [16], pancreas [17], and gastrointestinal tract [18]. In rats, mecamylamine-mediated blocking of the Chrna7 activity enhances inflammation severity [17]. In mice, the local administration of the agonist acetylcholine (ACh) into the spleen activates Chrna7. As a result of such activity, pro-inflammatory cytokines expression is inhibited, including tumour necrosis factor alpha (TNFα), interleukin-1 beta (Il1β), and interleukin-6 (Il6). Additionally, it affects the expression of releasing anti-inflammatory mediators such as interleukin-10 (Il10) and transforming growth factor β (TGFβ) [5]. Recent findings have also shown that, in diabetic mice, selective ablation of the Chrna7 promotes a low inflammatory activity mediated by peritoneal macrophages [19]. Altogether, these findings suggest conserved functions for cholinergic neurons in the immune response regulation. However, the functional relevance of Chrna7 during inflammation in lower vertebrates remains unexplored.

Additionally, equivalent molecule functions have been identified in ectothermic vertebrates, including fish. For example, nicotinic acetylcholine receptor transcripts have been detected in teleosts, including the zebrafish (*Danio rerio*) [20] and fugu (*Takifugu rubripes*) [21]. In rainbow trout (*Oncorhynchus mykiss*), Drescher and colleagues cloned the ACh receptor from saccular hair cells [22]. In zebrafish, two paralogs of the *chrna7* gene have been identified. In addition, a 1716 base pair (bp) in length transcript encoding a 509 amino acids protein is strongly expressed in different organs such as the brain, stomach, heart, muscle, and gonads [20]. Chrna7 also expresses in rainbow trout macrophages [23] and plays a role during viral infection in the zebrafish [2]. Other inflammatory reflex components, including acetylcholinesterase (AChE), have been detected in mononuclear cells from tilapia [24]. This enzyme catalyses the breakdown of ACh and of some other choline esters that function as neurotransmitters [25]. However, how cholinergic components function in the inflammatory reflex in teleost fish is still uncertain.

Unlike mammals, ectotherm organisms lack natural thermogenesis and therefore elevate their body temperature by moving to a warmer place [2,7]. Behavioural fever promotes organismal survival by enhancing protective mechanisms [26,27,28,29,30,31,32], including fever in endotherms [9]. In ectotherms, however, the underlying regulatory mechanisms triggered during behavioural fever are not well understood to date. Cumulative findings show that increased temperature during behavioural fever correlates with an acute immune response [2,7,8]. How thermal coupling influences the neural–immune system interaction and the cholinergic pathway activation during inflammation remains unclear in mobile ectotherms.

We combined temperature-dependent behaviour, gene expression, and functional analysis during behavioural fever to identify the regulators controlling the inflammatory reflex in fish. Specifically, we aimed to determine the defence response executed by the neural–immune axis during infection. Our data describe, for the first time, the molecular underpinnings that control the inflammatory reflex network in ectotherms. We identified cholinergic neuronal components and anti-inflammatory factors as crucial components of the neural–immune circuit, protecting from infection in ectotherms.

## 2. Results

### 2.1. Viral Infection Influences a Behavioural Fever Mechanism in Mobile Ectotherms

**Figure 1:**
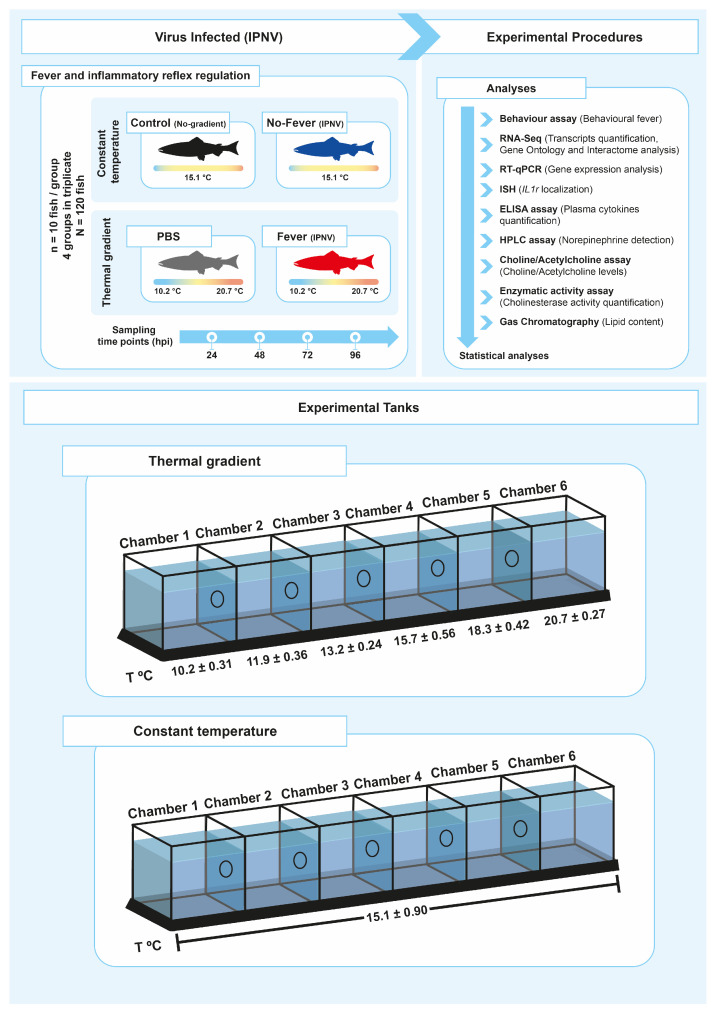
Experimental setup. Schematic representing the experimental design of thermal treatments after viral challenge. A summary of all analyses performed to study the neuro-immune interaction in the control of inflammation during behaviour fever in fish is presented (**A**). The experimental tanks set-up and the temperature associated with each chamber are also shown (**B**). The mean temperature ± SD value for each chamber is indicated. IPNV: infectious pancreatic necrosis virus; hpi: hours post-infection; SD: standard deviation.

**Figure 2:**
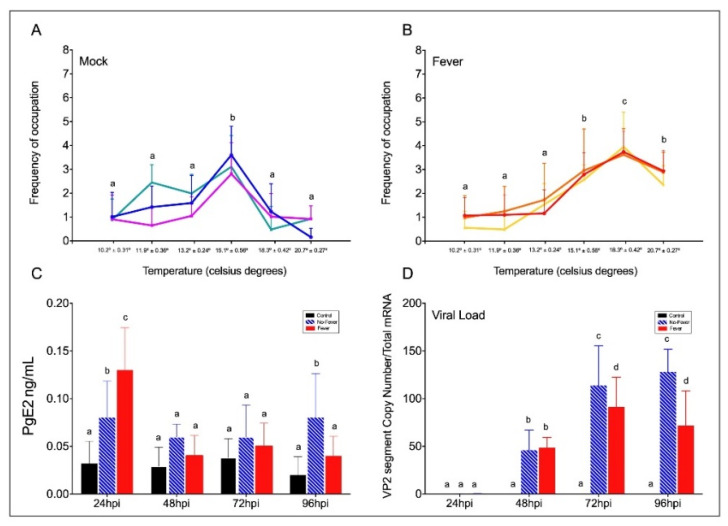
Behavioural fever in IPNV-challenged *Salmon salar*. (**A**) Frequency of chamber occupation in non-challenged individual fish (mock individuals = non-infected group in thermal gradient tank) over the 96 hpi period. The blue, green, and purple lines represent the frequency of occupation in each chamber by each technical replicate; mock (*n* = 3 technical replicates, mean ± SD). (**B**) Frequency of chamber occupation of IPNV-challenged individual fish. The red, yellow, and orange lines represent the frequency of occupation in each chamber by each technical replicate of fish challenged with IPNV under thermal gradient tank (*n* = 3 technical replicates, mean ± SD). (**C**) Plasma concentration of PgE2 (ng ml^−1^) in IPNV-infected fish. Bar graph showing differences between control (uninfected fish, black bars), no-fever (dashed blue bars), and fever (red bars) individuals (mean ± SD, two-way ANOVA followed by Tukey’s post hoc test, *p* < 0.0001). Different letters denote significant differences. (**D**) Viral load expressed as the abundance of VP2 segment (targeted with primer WB117) of the IPNV detected by RT-qPCR in the spleen. Bar graphs showing the differences between control (uninfected fish, black bars), no-fever (dashed blue bars), and fever (red bars) individuals (mean ± SD, two-way ANOVA followed by Tukey’s post hoc test, *p* < 0.001). Different letters denote significant differences. The mean and SD value for the copy number of the VP2 segment transcript per ng of total RNA is shown. IPNV: infectious pancreatic necrosis virus; hpi: hours post-infection; SD: standard deviation.

Behavioural fever manifests in response to pathogen challenge, including viral infection. To address whether aspects of behavioural fever are activated in fish as an immune response, we exposed salmon parr to an IPNV challenge under thermal treatment. All fish were infected at 12 °C for 96 h (enough time for the virus to initiate the infection) (Figure 1). Uninfected fish that were kept in a range of 10–20 °C in multi-chamber tanks showed that most of them preferentially distributed in the compartments ranged between 13 and 15 °C (chambers 3 and 4) (Figure 2A and Supplementary Figures S1A and S2A). The virus-infected individuals shifted their thermal preference and moved to warmer temperatures ranging between 18 and 20 ºC (chambers 5 and 6) over a 96 h period (Figure 2B and Supplementary Figures S1B and S2B). Statistical data analysis from three replicates showed that virally infected individuals manifested behavioural fever, as revealed by a significantly higher number of fish moving toward 18.3 ± 0.42 °C (chamber 5, Figure 2A,B; Wald test *p* < 0.001). The behavioural analysis performed (Wald’s test) differentiates preferential occupation for each chamber between the experimental groups. The analysis showed that individuals with access to temperature (fever group) spent significantly more time in chamber 5 (18.3 °C), unlike what was observed in mock-infected individuals. Phenotypic analysis performed on infected fish showed no physical abnormalities. This result validates, under viral infection, the development of a robust and specific behavioural fever response in fish.

In fish, behavioural fever is driven by peripheral inflammatory cytokines and PgE2 synthesis in response to pathogens [8]. We next examined the levels of PgE2 in control and viral-challenged salmon with access to the thermal gradient. We found that at 24 hpi, PgE2 synthesis was significantly higher in virus-infected individuals with access to the thermal gradient than in those infected but under limited thermal choice (Figure 2C). Then, it decreased in both treatments. This concentration pattern has previously been observed, and PgE2 was suggested to be a key precursor of behavioural fever in fish [2,8]. To investigate the temperature-dependent effect of behavioural fever on viral replication, we evaluated IPNV load over time in infected individuals. Quantitative analysis of the VP2 segment mRNA, as a readout of viral load (Figure 2D), revealed that fever significantly decreased systemic replication of the virus (significant at 72 and 96 hpi; two-way ANOVA *p* < 0.001). These data show a strong association between infection and temperature choice and reveal a behavioural fever mechanism driving viral clearance in ectotherms.

### 2.2. An Inflammation Activation Threshold Is Modulated by Behavioural Fever

**Figure 3:**
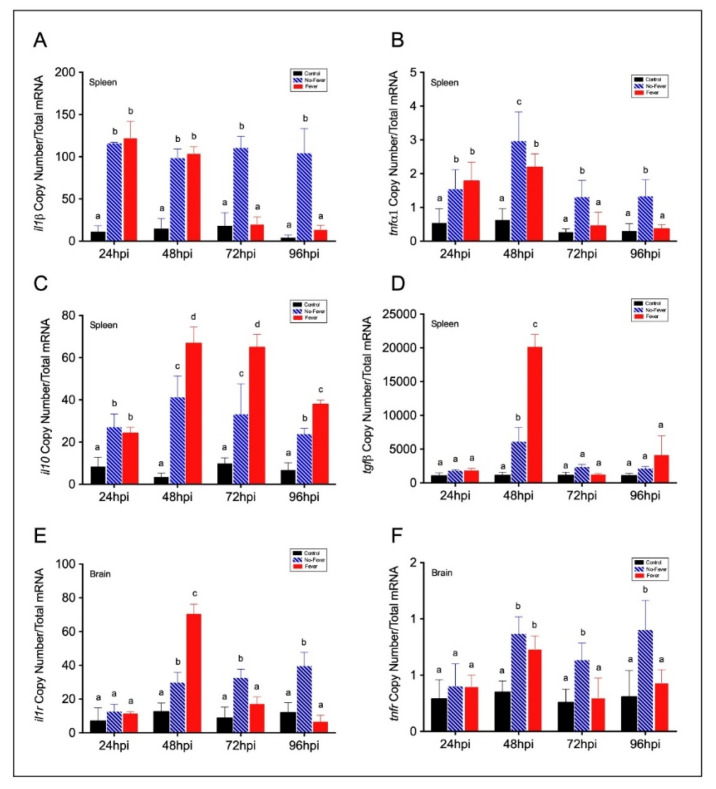
Expression profiles among pro- and anti-inflammatory factors in IPNV-infected fish spleen and brain. (**A**–**D**) Bar graph showing differences of inflammatory mRNAs abundance between control and IPNV-infected individuals under the thermal treatments. Expression of spleen pro-inflammatory *il1β* (**A**) and *tnfα1* (**B**) and anti-inflammatory *il10* (**C**) and *tgfβ* (**D**) transcripts in IPNV-challenged fish is shown. (**E**,**F**) Transcript expression profiles of neural inflammatory cytokines receptors *il1r* (**E**) and *tnfr* (**G**). Bar graphs showing the differences between control (uninfected individuals, black bars), no-fever (dashed blue bars), and fever (red bars) individuals (mean ± SD, two-way ANOVA followed by Tukey’s post hoc test, *p* < 0.01). Different letters denote significant differences. The mean and SD value for the copy number of inflammatory mediators and receptors mRNAs per ng of total RNA is shown. IPNV: infectious pancreatic necrosis virus; hpi: hours post-infection; SD: standard deviation.

Although the mechanistic basis for behavioural fever has been previously described in ectotherms [8], these fail to explain how temperature changes regulate inflammation over an extended time. Thus, once we identified a potential thermoregulatory mechanism mediating the response-specific inflammatory factors expression under the viral infection, we investigated the contribution of fever in this process. To address this hypothesis, we adapted the IPNV immersion challenge to salmonid fish under the thermal regimes described above for 5 days post-viral infection. We characterized in detail markers of the inflammatory response in the whole brain, spleen, and plasma of infected individuals sampled each 24 hpi. By performing RT-qPCR and ELISA assays, we analysed pro-inflammatory cytokine profiles. After infection, the mRNA abundance of pro-inflammatory cytokines *il1β* (Figure 3A) and *tnfα1* (Figure 3B) showed a significant difference between no-fever and fever individuals at 72 and 96 hpi (*p*-value *p* < 0.001; two-way ANOVA). Specifically, there were no significant differences in the *il1β* copy numbers between no-fever and fever groups at 24–48 hpi. After 48 hpi, *il1β* mRNA abundance significantly decreased in the behavioural fever group (Figure 3A). Alternatively, *tnfα1* transcript increased up to reaching a significant peak at 48 hpi in the no-fever individuals. However, after this time point onward it dramatically decreased in the fever group, and no significant differences were found between these groups prior to this time point (Figure 3B). The *il1β* and *tnfα1* cytokines mRNAs expression patterns were detected only in individuals that developed behavioural fever, in stark contrast to what was found in the no-fever group. This group showed a post-infection inflammatory response that was maintained through time. The observed differences can be due to the effect of temperature on the kinetics of the reactions; however, functional studies must elucidate the molecular bases of this regulatory mechanism.

To further identify the neural regulatory mechanism of inflammation, we tested the contribution of increased body temperature to this response by evaluating the expression of recognized pro- and anti-inflammatory factors and inflammatory cytokine receptors. We found that *il10* in spleen showed marked differences between no-fever and fever conditions at 48, 72, and 96 hpi (Figure 3C). Simultaneously, the measured abundance of anti-inflammatory cytokines *il10* and *tgfβ* highlighted significant differences at 48 hpi (Figure 3C,D). In no-fever individuals, the absolute copy number of measured *il10* cytokine mRNAs in the spleen increased slightly at 48 and 72 hpi (Figure 3C). A significant interaction between treatment and time was interpreted as a reduction of *tgfβ* transcript in the no-fever group after 48 hpi (Figure 3D, Tukey’s post hoc test *p* < 0.001). In the brain, a significant expression of the inflammatory receptor interleukin-1 (*il1r*) was detected at 48 hpi in the fever individuals, while at 72 and 96 hpi it was reduced (Figure 3E). In situ hybridization analysis showed that *il1r* transcripts were specifically localized in the telencephalon and optic lobes, suggesting a spatially restricted neural inflammatory response (Supplementary Figure S4). In contrast, the *tnfα1* receptor (*tnfr*) mRNA abundance was higher in the no-fever group after several hours post-infection (Figure 3G).

**Figure 4:**
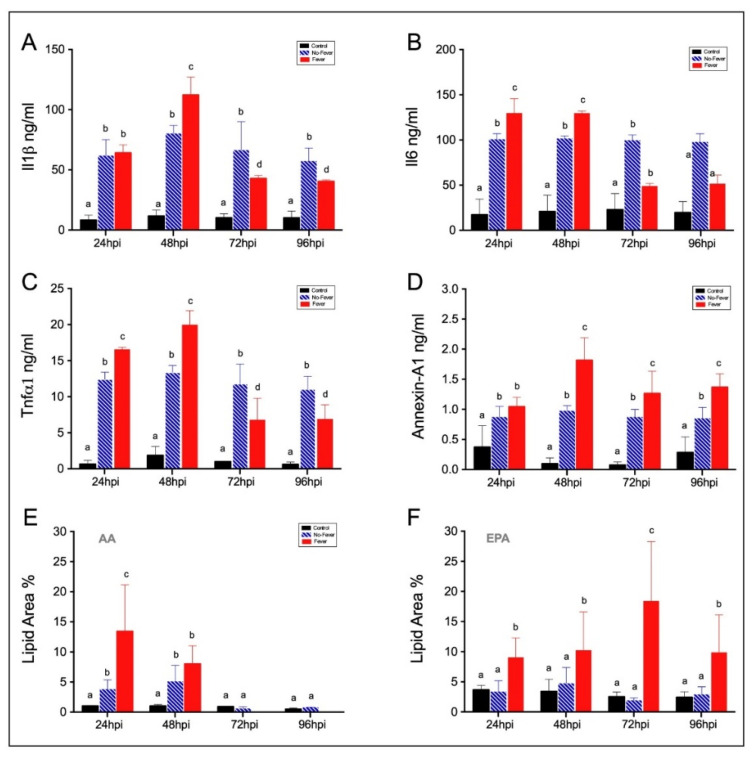
Indirect ELISA detection of pro- and anti-inflammatory cytokines plasmatic release and hepatic polyunsaturated fatty acids in response to IPNV challenge. (**A**–**D**) Bar graphs showing the expression of Il1β (**A**), Il6 (**B**), Tnfαr (**C**), and annexin-1A (**D**) proteins between groups subjected to thermal gradients. The differences between control (uninfected individuals, black bars), no-fever (dashed blue bars), and fever (red bars) fish are shown (mean ± SD, two-way ANOVA followed by Tukey’s post hoc test, *p* < 0.0001). Different letters denote significant differences. (**E**) Arachidonic acid (AA) and (**F**) eicosapentaenoic acid (EPA) hepatic profiles (% of total fatty acid identified in the liver) in fish after IPNV infection. Bar graphs showing the differences between control (uninfected fish, black bars), no-fever (dashed blue bars), and fever (red bars) individuals. Different letters represent significant differences (mean ± SD, two-way ANOVA followed by Tukey’s post hoc test, *p* < 0.05). The cytokine ELISA assay is described in the Supplementary Materials section. ng/mL: nanograms per millilitre; hpi: hours post-infection; SD: standard deviation.

To determine whether behavioural fever shifts the global defence response by either enhancing or decreasing the inflammatory pathway, we performed measurements of plasma levels of Il1β, Il6, and Tnfα1. A significant increase in plasma levels of the inflammatory cytokines was identified in the fever and no-fever groups, in contrast to what was observed in control individuals (Figure 4A–C; *p* < 0.01). Remarkably, during behavioural fever at 48 hpi, Il1β (Figure 4A), Il6 (Figure 4B), and Tnfα1 (Figure 4C) protein levels were higher than those in the no-fever individuals. Measurement of plasma levels of a recognized anti-inflammatory mediator annexin-1A suggests immunosuppression action in infected individuals (Figure 4D; *p* < 0.005). The registered values of annexin-1A in the fever group are similar to those registered in functional studies performed with salmon infected with IPNV, showing the robustness of the presented results. The literature shows that in fish, as in mammals, the release of annexin-1A is mediating the anti-inflammatory actions through inhibition of the action of glucocorticoids [33]. In this sense, annexin-1A levels in the fever individuals and decreased plasmatic concentration of pro-inflammatory mediators indicate the role of behavioural fever in the regulation of the inflammatory response.

To further correlate PgE_2_ synthesis with substrates of inflammatory mediators, we evaluated changes in the content of arachidonic acid (AA) and eicosapentaenoic acid (EPA) in liver samples. Under the fever condition, AA levels were significantly higher in the liver during the first 24 and 48 hpi (Figure 4E,F) (two-way ANOVA; *p* < 0.001). Specifically, in fever individuals, we found a significant increase of AA, reaching 13.9% and 7.6% of the total lipid content at 24 and 48 hpi, respectively, followed by sudden reduction (Figure 3E; *p* < 0.001). Statistical comparison of the EPA profile between IPNV-infected fish, showed an increase of 18.4% and 9.2% of the total lipid content in the fever group at 24 and 48 hpi. This content reached the highest level at 72 hpi (Figure 4F; *p* < 0.05), while it was significantly higher than the levels observed in no-fever individuals at all time points. The canonical reduction of inflammatory cytokines and other pro- and anti-inflammatory mediators observed in the fever group, reveal that the inflammatory response is dependent on temperature-shift in mobile ectotherm organisms.

### 2.3. Behavioural Fever Initiates a Neural–Immune Regulation of Inflammation

**Figure 5:**
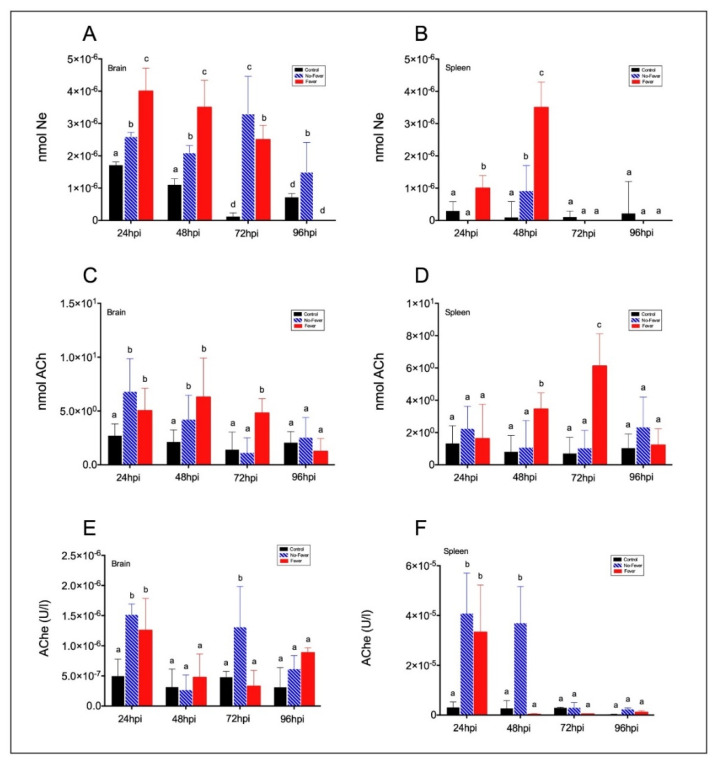
Behavioural fever increases norepinephrine and acetylcholine levels in the brain and spleen of viral-infected fish. (**A**,**B**) Norepinephrine (Ne) was measured by HPLC-FL system in brain and spleen homogenates. (**A**) In the brain, Ne levels were significantly reduced in no-fever as compared with fever fish. Different letters denote significant differences (mean ± SD, two-way ANOVA followed by Tukey’s post hoc test, *p* < 0.001). (**B**) In the spleen, Ne content was altered in the febrile state as compared with no-fever individuals, thus revealing a thermo coupled effect of virus infection in Ne levels in the target organ (two-way ANOVA followed by Tukey’s post hoc test, *p* < 0.0001). (**C**,**D**) Acetylcholine (ACh) levels were measured by colorimetric ELISA in homogenates from the fish brain (**C**) and spleen (**D**) during viral challenge in fever and no-fever conditions. Results are expressed as the mean of two independent experiments (mean ± SD, two-way ANOVA followed by Tukey’s post hoc test, *p* < 0.05). Different letters denote significant differences. Acetylcholinesterase (AChE) activity (μmol L^−1^min^−1^) in the brain (**E**) and spleen (**F**) of viral-infected individuals was significantly reduced in febrile state. Bar graphs showing differences between control uninfected (black bars), no-fever (dashed blue bars), and fever (red bars) individuals (mean ± SD, two-way ANOVA followed by Tukey’s post hoc test, *p* < 0.05). Different letters denote significant differences. nmol: nanomoles; U/L: units per litre. hpi: hours post-infection; SD: standard deviation.

In mammals, it is known that anti-inflammatory reflex molecules can inhibit systemic pro-inflammatory cytokine synthesis and leukocyte migration at sites of organ injury during inflammation and febrile episodes [9]. However, the molecular nature of factors regulating the inflammation during febrile episodes in ectotherms has not been identified. In addition, previous studies suggest a central role of a cholinergic pathway in regulating systemic immune responses [13,34]. Under the infection, Ne neurotransmitter showed a significant increase at 24, 48, and 72 hpi and a decrease at 96 hpi (Figure 5A; *p* < 0.01), while a significant increase was detected at 48 hpi in the spleen of the fever-infected group (Figure 5B; *p* < 0.001). We also found high levels of ACh at 72 hpi (Figure 5C,D; *p* < 0.0001) but low activity of AChE (Figure 5E,F) in the fish spleen. In contrast, in the no-fever group, the ACh levels were significantly lower at 48 and 72 hpi (Figure 5D). The detected activity of AChE in the fever group indicates that it was not degraded in the target tissue during behavioural fever, and this molecule could become available to interact with Chrna7, thus inhibiting the release of inflammatory factors.

**Figure 6:**
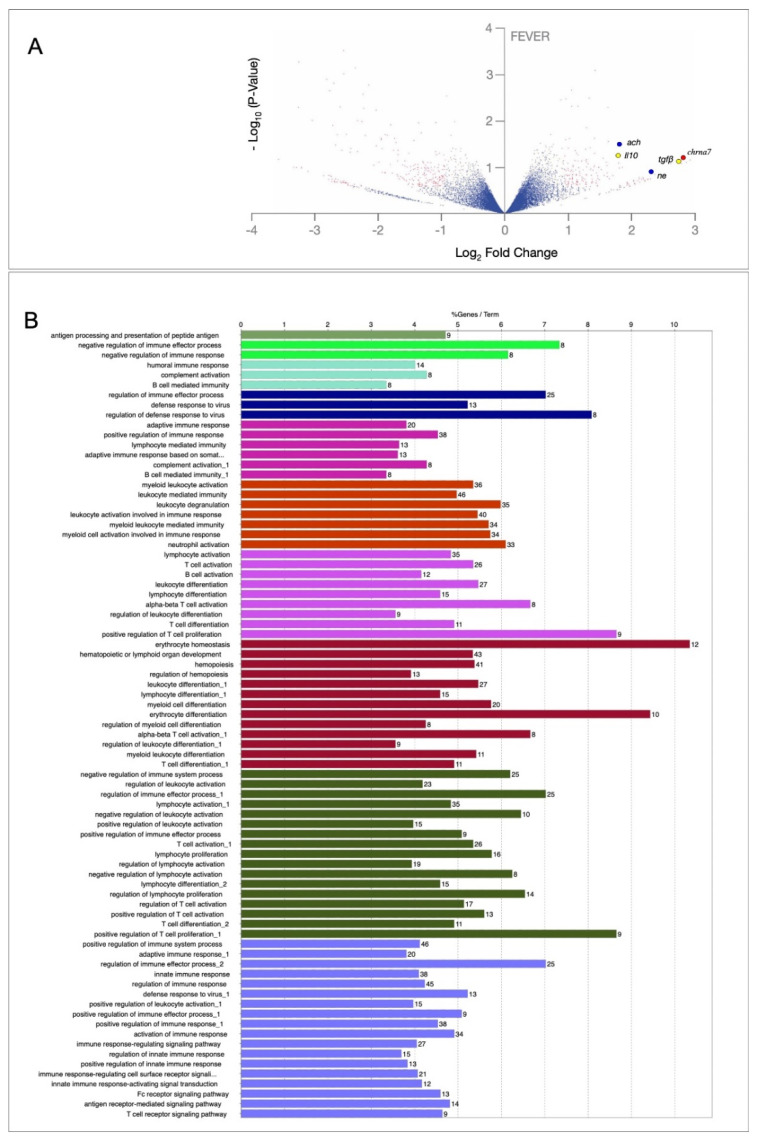
Transcription-wide RNA-Seq screen identifies key inflammatory reflex genes during behavioural fever. (**A**) Volcano plot showing transcripts significantly enriched in spleen following fever induction. Red small dots indicate genes with known functions associated with immune and inflammatory responses and a significant fold change over the background values. Blue, yellow, and red circles highlight members of the inflammatory reflex complex that were significantly enriched such as *ache* and *ne* (blue)*, tgfβ* and *il10* (yellow), and the *chrna7* (red) transcripts. (**B**) Gene ontology enrichment analysis of 761 differentially expressed transcripts (GO-DAVID and ClueGo Cytoscape Plugins) showing the set of immune-related gene functions playing a role during behavioural fever.

To explore how the febrile state drives specific changes in gene expression, we profiled the transcriptome in fish spleen under fever conditions using RNA-Seq (Figure 6A). We selected 24 h, which is the sample time with the higher inflammatory activity. A total of 761 DEGs were obtained between all experimental conditions (DGE 24 hpi; log_2_ fold change ≥ |2|; *p*-value ≤ 0.05). Clustering analysis (ClueGO) was used to examine the specific gene expression modules expressed in the fever fish group. These gene clusters were correlated with expression patterns reflecting coherent inflammatory or immunological functions. We selected these modules with over-representation of node–node interactions (immune enrichment analysis) that were found for each condition (fever versus no-fever) and conducted GO functional analysis with GO-DAVID [35] and the ClueGO Cytoscape plugin [36]. By using these bioinformatic tools, we predicted a significant enrichment of functional GO clusters related to the immune response (Figure 6B).

We identified a strong functional enrichment of specific immune response categories in the fever group associated with a gene set as a whole. We also found the enrichment of negative regulators of the immune response (*p* = 0.01, *q* = 0.05), antigen processing and presentation of peptide antigen (*p* = 0.05), and T cell and leukocyte activation (*p* < 0.02; proportion of false discoveries at this *p*-value threshold, *q* = 0.30). Statistical analysis confirmed that thermal ranges modified the fish transcriptome, forming clusters of transcripts with different expression patterns. Remarkably, our results of RNA-Seq in spleen of the fish showed that genes related to the inflammatory arc circuit, such as *ach* and *ne* neurotransmitters, as well as anti-inflammatory cytokines *tgfβ, il10,* and the *chrna7* were up-regulated in the fever group (Figure 6A). We found that the largest module (24% of the genes; mean r = 0.51) consisted of B cell activation, positive regulators of T cell proliferation, and erythrocyte homeostasis gene enrichment (*p* = 0.001, *q* = 0.06). RNA-Seq and GO analysis allowed us to identify behavioural fever as the cue driving specific immunological response.

**Figure 7:**
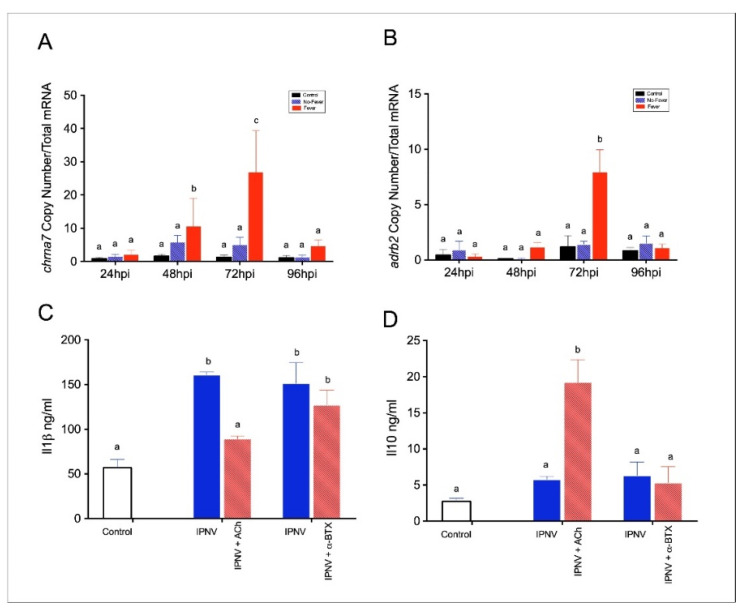
Expression profiles among neuroregulatory receptors. (**A**) *adrb2* and (**B**) *chrna7* in spleen of IPNV-challenged individuals. Bar graphs showing significant differences in mRNA abundances between control uninfected (black bars), no-fever (dashed blue bars), and fever (red bars) individuals at 48 and 72 hpi (mean ± SD, two-way ANOVA followed by Tukey’s post hoc test, *p* < 0.05). The mean and standard error (SD) value for the copy number of *chrna7* and *adrb2* transcripts per ng of total RNA is shown. Different letters denote significant differences. (**C**,**D**) Indirect ELISA detection of pro- and anti-inflammatory cytokine release in spleen cells treated with *α*-BTX and ACh prior to IPNV stimulation. Cytokine Il1β (**C**) and Il10 (**D**) factors release was significantly different between IPNV + ACh-treated cells compared with IPNV-only-treated cells. Bar graphs showing the differences between control uninfected (white bar), IPNV (blue bars), and IPNV + ACh or *α*-BTX (dashed red bars) individuals (*n* = 10) (mean ± SD, two-way ANOVA followed by Tukey’s post hoc test, *p* < 0.5). Different letters represent significant differences. ng/mL: nanograms per millilitre; IPNV: infectious pancreatic necrosis virus; hpi: hours post-infection; SD: standard deviation.

In correlation with our transcriptomic data, we also found a significant increase of *adrb2* (Ne receptor) and *chrna7* (ACh receptor) expression after infection (Figure 7A,B). The relationship between these differentially expressed neurotransmitter–receptor complexes reveals a cholinergic anti-inflammatory pathway as the potential mechanism promoted by behavioural fever. Additionally, we tested the ability of agonists and antagonists of Chrna7 to alter the neural circuit and shed light on its functional properties during viral infection. We also aimed to dissect the role of the receptor in the inflammatory response through a functional pharmacological study. To do this, we used a selective α7 nicotinic ACh receptor agonist and the antagonist α-bungarotoxin (α-BTX). In mammals, the selective agonist ACh binds to Chrna7 and inhibits the release of inflammatory mediators [37]. In this study, ACh induced the inhibition of inflammatory factors such as Il1β at 24 h post-incubation (Figure 7C), as well as promoted the release of the anti-inflammatory cytokine Il10 in spleen macrophages challenged with IPNV (Figure 7D). On the other hand, the cholinergic receptor antagonist α-BTX blocks Chrna7 action in rats [38,39] and fish [23]. At 24 h post-incubation, we did not detect significant differences in Il1β and Il10 protein levels after a challenge with IPNV and α-BTX (ordinary one-way ANOVA; F_(2,64)_ = 3.83, *p* ≤ 0.01). These findings support the notion that spleen macrophages might utilize the Chrna7-mediated mechanism regulating pro- and inflammatory cytokine release.

**Figure 8:**
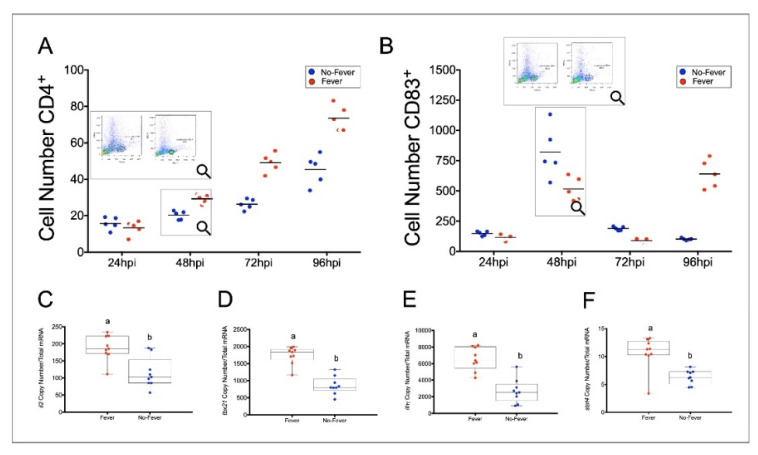
Behavioural fever promotes immune response activation by increasing spleen CD4+ T cell number and lymphocyte-related immune gene expression. (**A**) Spleen-derived lymphocyte population of CD4+T cells (number of cells positive immunodetected with the CD4-BV421 antibody) in no-fever (blue circles) and fever individuals (red circles). The subpanel shows that at 48 hpi, individuals displaying behavioural fever exhibit higher proportions of CD4+ T cells in the spleen (*p* = 0.031, *n* = 60; *y* axis shows the T cells populations after IPNV challenge and the number of positive cells identified by the CD4-BV421 antibody. (**B**) Number of CD83+ positive cells (number of positive cells detected by the CD83-BV421 antibody) in the spleen of viral-infected individuals. No-fever (blue circles) and fever (red circles) fish are shown. The subpanel shows that, at 48 hpi, the no-fever individuals exhibit a higher number of CD83+ positive cells (*p* = 0.031, *n* = 60; *y* axis shows the percentage of T cell populations after IPNV challenge. Flow cytometry plots illustrating the cell populations of interest (delineated by black lines) are shown as inserts. (**C**–**F**) Expression profiles among lymphocyte-related immune genes detected at 24 hpi. Box plots show the analysis of *il2* (**C**)*, tbx21* (**D**)*, ifnγ* (**E**), and *stat4* (**F**) transcripts abundance in response to thermal treatments. The mean and standard error (SD) value for the copy number of lymphocyte-related transcripts per ng of total RNA is shown. Different letters denote significant differences between fever (red squares) and no-fever (blue squares) individuals (mean ± SD, two-way ANOVA followed by Tukey’s post hoc test, *p* < 0.01). hpi: hours post-infection; SD: standard deviation.

To identify the lineage of cytokine-producing cells, we estimated the proportion of the two-main cell populations, CD4+ T and CD83+ T cells, in behavioural fever-induced individuals. Our FACS analysis showed that the no-fever group had a low proportion of CD4+ T cells in the fish spleen (Figure 8A). In contrast, the fever group displayed a significantly increased number of these cells after 48 hpi (Figure 8A, *n* = 10; *p* < 0.05). In mammals, CD83 is a marker of mature dendritic cells (DCs), such as Langerhans, in the skin, and peripheral blood cells. High surface CD83 expression is found in mature DCs after exposure of the immature ones, which is induced after pathogen attack [40]. In this study, flow cytometry of whole spleen leukocytes, double-stained with anti-CD4-1 and anti–CD83 mAbs, show that the CD83+ population remained relatively invariable in the fever individuals compared with those of the non-infected group after IPNV infection (Figure 8B; *n* = 10, *p* > 0.05). Fever and infected individuals were compared with infected fish not exposed to behavioural fever. To further characterize the CD4+ spleen lymphocyte phenotype and confirm their identity as T cells, we assessed the expression of a variety of Th1 cell-related genes at 24 hpi. As expected, transcripts defining CD4+ Th1 cells identity were over-expressed in fever group individuals. Furthermore, no-fever individuals expressed low mRNA abundance of key Th1 cell marker genes (interleukin 2 (*il2*), t-box transcription factor 21 (*tbx21*), interferon γ (*ifnγ*), and signal transducer and activator of transcription 4 (*stat4*)) (Figure 8C–F). 

## 3. Discussion

Inflammation is a complex physiological response central to the development of successful immune protection. Without this fundamental biological strategy, effective prevention against cellular damage and infection results in immunity impairment. Inflammatory response acquisition depends on pathogen recognition and recruitment of immune cells to return to normal homeostasis [41]. However, the mechanisms that regulate inflammation after pathogen infection have not been fully understood in ectothermic vertebrates. The data presented here show that behavioural fever is modulating the inflammation through the activation of neural components of the cholinergic arc. This mechanism has been previously suggested in the regulation of cytokine production in fish [23]. Here, we have found that neural components of the cholinergic arc act in synergy with behavioural fever. The results also highlight a spatiotemporal genetic control of neuro-immune communication during inflammation in fish.

Fever and defence response are generated by a highly conserved set of pathogen recognition receptors (PRR) [41]. This activation leads to significant development of local and systemic defence responses, including inflammatory cytokines production [42]. The pathogen–PRR interaction leads to the synthesis of pro- and inflammatory cytokines [42,43,44]. Specifically, the pathogens break down the delicate balance of the inflammatory response by promoting a positive feedback in immune cells. Additionally, this triggers the upregulation of several factors, including cytokines Il1β, Il6, and TNFα. In this study, we examined the expression of the *Salmo salar* tumour necrosis factor alpha-1 (*tnfα1*) mRNA to explore the inflammatory response [45]. In our studies, behavioural fever promoted suppression of the release of inflammatory cytokines Il1β, Il6, and TNFα1 and the inhibition of other inflammatory mediators such as AA and PgE2. AA acts as a precursor of PgE2, which plays an essential role in inflammation. Additionally, it is considered a fundamental mediator of fever in the preoptic area of the brain [8,46]. Thus, our analysis highlights the importance of temperature shifts in the identification and accuracy of the immune response at both the physiological and molecular levels.

In mammals, inflammation during pathogenesis frequently results in signs such as hypo- or hypertension and oedema and can eventually cause organ failure and death [47]. Increasing evidence highlights how the inflammatory response should be monitored, integrated, and controlled by the nervous and immune cells, thus describing a tight interaction between these essential systems [48,49]. The coupling between the nervous system and fever consists of systemic AChE modulation and the expression of cholinergic receptors in immune cells, thus triggering the activation of the inflammatory reflex. The cholinergic inflammatory pathway uses the neurotransmitters ACh and Ne to interact specifically with the Chrna7 on immune cells [50]. The receptors respond to neurotransmitters by generating connections with lymphocytes or macrophages and the vagus nerve, which results in the suppression of pro-inflammatory cytokines [13]. We found that when the fish display behavioural fever, it promotes extensive and highly specific temperature-dependent regulation of neurotransmitters in the brain and other organs. Specifically, we showed a significant effect under temperature-changing conditions, where neurotransmitters such as ACh and Ne, as well as neuroregulatory receptors within the defined cholinergic arc (Chrna7 and Adrb2), were overexpressed. Additionally, under fever condition, the AChE activity is inhibited.

Remarkably, we also observed the release of anti-inflammatory cytokines such as Il10 and Tgfβ, which have been described as key components of the inflammatory reflex network [5]. These factors participate in either stimulating the Th1 or suppressing the T helper type 2 (Th2) immune response through the involvement of CD4+ regulatory T cells (Treg) [51,52]. We found a significant increase in CD4+ cells in the spleen of infected individuals developing behavioural fever. Remarkably, our results also show differential gene expression of *il2*, *tbx21, ifnγ*, and *stat4* in spleen cells. These support the idea that at least within the measured time scales, a Th1-driven response is executed [53,54,55]. A Th1-like response has been previously reported in fish under viral infection [56] and behavioural fever [2,8]. This response also participates in the regulation of inflammation [57], highlighting the relevance and strength of our findings.

The release of Il10 typically comes after the activation of the inflammatory response to decrease inflammation and helps overcome damaging effects of cytokine dysregulation. CD4+ Tregs cells play a key role in the regulation of inflammation through the release of anti-inflammatory mediators such as Il10. However, others cell types such as monocytes/macrophages are also important sources of this cytokine during pathogenesis [52,58,59]. Some non-immune cells such as keratinocytes or epithelial cells can also produce Il10 [60]. In our study, we used a heterogeneous group of spleen cells that, under viral infection and behavioural fever, decreased the inflammatory state by releasing Il10 and Tgfβ. In addition, we isolated spleen macrophages to develop functional studies. Under these experimental conditions, these cells could release anti-inflammatory mediators. However, the actual local or systemic contribution of a specific subset of spleen cells in the regulation of inflammation was not determined. Our pharmacological assay, carried out with the acetylcholine receptor antagonist α-BTX, revealed a key role of Chrna7 in the inflammatory reflex. Functional studies in trout have shown that the residues involved in the α-BTX-Chrna7 binding are conserved [23]. This indicates that the receptor would be able to bind α-BTX with high affinity in salmon species. In our study, Chrna7 binds to α-BTX, which inhibits the release of Il10 splenic macrophages of fish challenged with the virus. Similar results have been described in trout [23] and mammal macrophages [12]. The concentration spectrum used in our study was similar to one previously described [12,61]. These are considered physiological doses used on functional studies to get insights into the inflammatory reflex activation.

**Figure 9:**
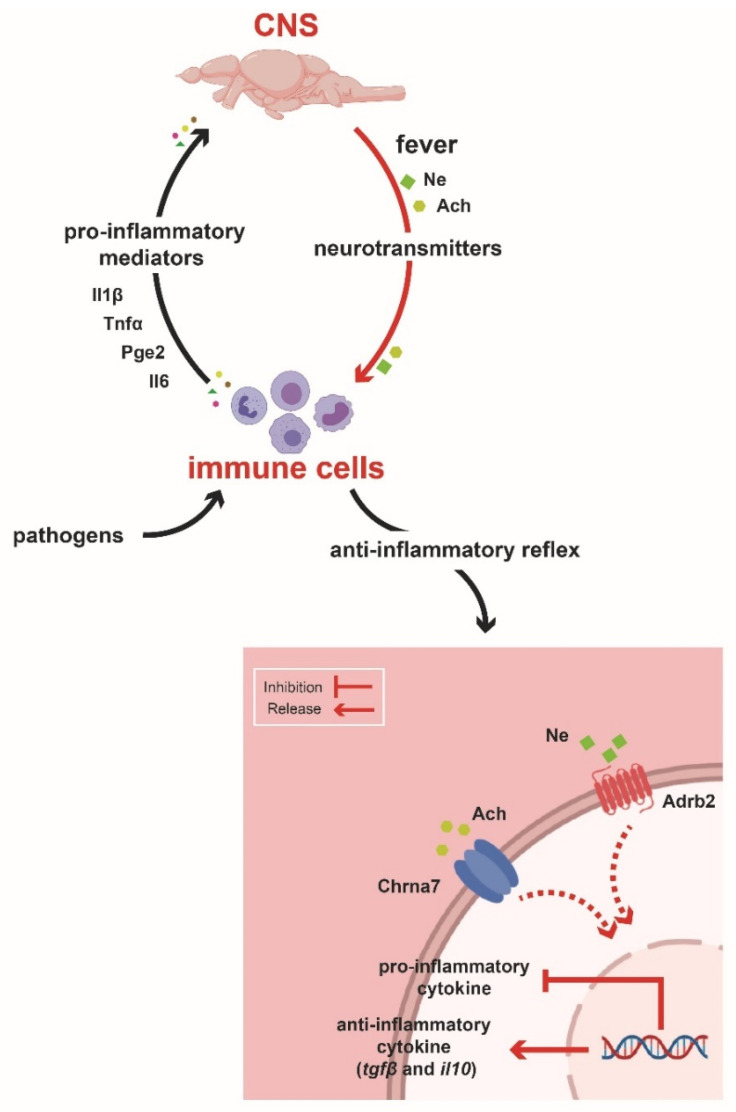
Working model of the Immune–Sensory–Neuron Interface during behavioural fever. Working model based on the results obtained in the present study. We propose that pathogens or organ injury results in the release of inflammatory mediators, such as pro-inflammatory cytokines Il1β, Il6, Tnfα, and PgE_2_, by immune cells. These signals act on cytokine receptors expressed on peripheral axonal terminals of sensory neurons in the central nervous system (CNS). The release of PgE_2_ can directly activate behavioural fever [7]. Simultaneously, the neuronal activation by the pro-inflammatory stimuli promote the release of neuropeptides, such as ACh and Ne, which in turn modulates inflammatory responses. Specifically, ACh and Ne bind to the Chrna7 and Adrb2 receptors, respectively, which are expressed on macrophages and other immune cells. The neural–immune reflex triggers intracellular signalling pathways, activating anti-inflammatory genes transcription and suppressing the production of Tnfα and several other pro-inflammatory factors.

The immune response modulates inflammation via neuroregulatory mechanisms, such as the cholinergic inflammatory pathway. In this way, the positive feedback loop driven by dysregulation in cytokine production can be prevented in mammals [5]. Our results highlight the critical function of the brain in controlling the distinct phases of the inflammatory response by a regulatory mechanism induced by behavioural fever (Figure 9). Beneficial or harmful effects of this adaptive trait have been proposed in ectotherms [7,29]. These studies extend our previous observations and suggest a crucial role of temperature changes in the regulation of inflammation. To our knowledge, the pathogen-mediated neuronal activation mechanism has not previously been studied in fish. Thus, our findings support the hypothesis that highly conserved molecular factors, also identified in mammals, might mediate the communication between the immune and neural systems during febrile states (Figure 9). Moreover, the aforementioned results highlight the relevance of behavioural fever in mobile ectotherms, particularly in response to an infection. These also suggest the involvement of the neural system driven by the immune reflex in the regulation of inflammation.

We propose that the activation of Chrna7 by cholinergic neurotransmitters regulates inflammation during behavioural fever in fish. Gene expression analysis supports the idea that this process induces a coordinated regulatory response at the transcriptional level, which is distinct to responses under limiting environmental conditions (no-fever group). Our findings highlight the influence of a thermo-coupled mechanism with neural regulation in preventing the harmful effects of inflammatory cytokines overexpression. To our knowledge, these novel results identify an unprecedented link between neurotransmitters, such as ACh and Ne, and the activation of Chrna7 to release the anti-inflammatory cytokine Il10 and a Tgfβ-mediated response during behavioural fever (Figure 9). These results also correlate with differentially expressed neurotransmitter–receptor complexes and our functional analysis. Thus, confirming that a cholinergic anti-inflammatory pathway might be a mechanism promoting the regulation of inflammation during behavioural fever. However, further functional assays are required to fully demonstrate this hypothesis. This effort may include loss-of-function studies of the Chrna7 by using genome editing technologies in a fish model (S.B. and R.F., unpublished data). One of the experimental limitations of the present study is related to the number of individuals used in the lipid profile analysis and the transcriptional analysis. However, despite these limitations, the present results are consistent; for example, we observed significant upregulation of genes related to the anti-inflammatory response pathway and few positively regulated heat-shock genes (Benjamini–Hochberg corrected *p* values <0.05). Furthermore, our data set’s number of repressed genes is similar to those obtained from previous behavioural fever studies [2,8]. These results suggest a specific activation of neural–immune regulation during behavioural fever rather than a heat-shock response.

## 4. Materials and Methods

### 4.1. Animal and Experimental Conditions

*Salmo salar* parr were purchased from AquaGen S.A. (Melipeuco, Chile), and they were maintained in accordance with the research guidelines for the use of laboratory animals established by the Chilean National Commission for Scientific and Technological Research (CONICYT) and authorized by the University of Concepcion Institutional Animal Care. The recirculating fish system (Animal Care) consisted of sterilized freshwater and a flow rate of 5 m^3^ per hour (hr). Water temperature was measured twice per day (Figure 1). Dissolved oxygen was measured daily and kept above 9 mg/L. Ammonia, nitrite, and pH were measured twice per week and kept under 0.05 mg/L, 0.01 mg/L, and 8.0 ± 0.5, respectively. A 12 h light/12 h dark photoperiod was used to artificially reproduce the autumn–winter, in correspondence with the annual cycle of species [62]. Water temperature, pH, and dissolved oxygen were measured using a Multiparameter Water Quality Meter 9829 (Hanna Instruments®, Woonsocket, RI, USA). Ammonia and nitrate/nitrite were measured using QUANTOFIX® test strips (MACHEREY-NAGEL, Dueren, Germany).

### 4.2. Infectious Pancreatic Necrosis Virus (IPNV) Assay

In vivo infection of salmon parr (121 ± 11.3 mg) with infectious pancreatic necrosis virus (IPNV) was performed by immersion using dechlorinated water as previously described [63]. Clarified supernatant from IPNV-infected CHSE-214 cell monolayers (10 × 10^5^ plate forming units (PFU)/mL) was added to tanks containing the fish (*n* = 120). After 120 min (min), fish were separated in two groups and placed in the two different experimental thermal set-ups: (a) constant temperature (mean temperature 15.1 ± 0.9 °C, “no-fever”) and (b) temperature gradient (mean temperature 15 ± 5.7 °C, “fever”). In parallel, fish in another tank (*n* = 120) were exposed to 100 mL of virus-free cell culture supernatant (control or mock-infected individuals). Four independent experimental infection assays were set-up: (1) control with no gradient, (2) fish exposed to 100 mL of virus-free cell culture supernatant under a temperature gradient (mock-infected individuals), (3) virus-infected individuals under a temperature gradient, and (4) infected individuals under constant normothermic conditions. The fish were euthanized by prolonged immersion in tricaine methane sulfonate (MS-222, 0.1 g/l, Sigma-Aldrich), and the brain, spleen, and plasma were sampled 24, 48, 72, and 96 h post-infection (hpi). The following phenotypes were scored on each fish: abdominal distension, exophthalmia, impaired swimming, and skin/fin base haemorrhages. In all groups, behavioural data were recorded as described below. RT quantitative PCR (RT-qPCR) of each sampled fish was used to estimate the IPNV load by targeting the viral segment virus protein 2 (VP2) using primers WB117 and Universal ProbeLibrary probes (UPL) as previously described [64,65,66]. All fish used in the experiment were identified as IPNV-positive using primers WB117 (Supplementary Table S1).

### 4.3. Behaviour Studies

The experimental thermal gradient was conducted as previously described [7]. The experimental fever tank was a 2.36 m^3^ tank (200 cm × 30 cm × 30 cm) divided with 5 transparent Plexiglas screens to create 6 equal interconnected chambers. Each screen had a hole in the centre (3 cm diameter and 10 cm distance from the bottom) to allow the connection between chambers. The Plexiglas screens were 1.5 cm thick, providing the necessary isolation between both adjacent chambers and avoiding temperature overlap. Three video cameras provided continuous monitoring of each tank chamber. A recording regime of 10 s every 15 min was used during daylight hours (96 h = 480 recorded measures). The distribution of fish into the six compartments was monitored over time with video cameras, and the number of fish in each compartment was counted manually from the images captured at each successive 15 min, resulting in 96 measurements per day. Temperature measurements were performed in each chamber twice daily. Thermal gradients were established with a mean differential temperature of 15 ± 5.7 °C from chamber 1 to chamber 6 by heating (chamber 6; mean temperature = 20.7 ± 0.27 °C) and simultaneously cooling (chamber 1; mean temperature = 10.2 ± 0.31 °C). All temperatures were recorded every day at the same time. Under all experimental conditions, thermal gradient or normothermic conditions, 3 groups of 10 fish were used for each treatment and were introduced into chamber 4 to provide a 12 h acclimation period. Experimental groups were those described in Section 4.2 (Figure 1). The mean number ± standard deviation (SD) of fish present in the chamber per day was registered for each experimental group. Two statistical analyses were performed to understand behavioural fever in the four experimental groups. The main aim was to evaluate whether fish challenged with IPNV preferred chambers with higher temperatures. An additional aim was to evaluate how fish were distributed across the 6 chambers and to determine how the challenge with IPNV affected this distribution. The data were analysed by using binomial generalized linear models; however, the dispersion statistics from these models suggested a variation in the data smaller than the expected variability based on the binomial distribution. Then, generalized Poisson regression models were used [67], which are recommended for under-dispersed data [68]. The number of fishes that were found in chambers 5 and 6 was modelled as a function of the two temperature groups (gradient and constant), challenge groups (control, mock, fever, no-fever), and their interaction (4 groups). Our preliminary results show that the different controls used did not differ significantly between all any of the analytical measures used. Therefore, to reduce the complexity of interpreting our results, we used just three experimental conditions to develop all analytical measures: 1) control with no gradient (control), 2) virally infected individuals under a temperature gradient (fever), and 3) virally infected individuals under constant normothermic conditions (no-fever).

### 4.4. RNA Extraction, cDNA Synthesis, and Transcript Quantification

Ten random fish were sampled for each treatment (control, fever, no-fever) and time (24, 48, 72, and 96 hpi) and were subsequently snap-frozen in liquid nitrogen and conserved at –80 °C. Total RNA was extracted from the brain and spleen of individual fish with the TRI Reagent® (0.5 mL; Sigma-Aldrich, St. Louis, MO, USA) and quantified by absorbance at 260 nm. Only samples with an A260/280 ratio between 1.8 and 2.1 and an A260/230 ratio above 1.8 were used for reverse transcription. Purified RNA integrity was confirmed by agarose-denaturing gel electrophoresis (>9 samples per treatment and time met the suggested quality standards). cDNA was synthesized from 50 µL of total RNA (200 ng/µL) using the RevertAid H Minus First Strand cDNA Synthesis Kit (Fermentas, Waltham, MA, USA) according to the manufacturer’s indications. RT-qPCR was performed using the StepOnePlus™ Real-Time PCR System (Applied Biosystems, Life Technologies, Carolina, USA), and each assay was run in triplicate using the Maxima SYBR Green qPCR Master Mix-2X (Bio-Rad, Carolina, USA). For qPCR assays, 5 μL of synthetized cDNA was diluted with 15 μL of nuclease-free water (Qiagen, Hilden, Germany). Each qPCR mixture contained SYBR Green Master Mix, 2 μL of diluted cDNA, 500 nmol/l each primer, and RNase-free water to a final volume of 10 μL. Amplification was performed in triplicate on 96-well plates with the following thermal cycling conditions: initial activation for 10 min at 95 °C, followed by 40 cycles of 15 s (s) at 95 °C, 30 s at 60 °C, and 30 s at 72 °C. A dilution series made from known concentrations of plasmids containing the PCR inserts was used to calculate absolute copy numbers for each examined gene (Supplementary Table S1). Gene expression and protein analysis between non-simulated controls (control) and mock individuals did not show significant differences (unpublished data).

### 4.5. In Situ Hybridization

Il1 receptor transcript was detected in the brain by chromogenic in situ hybridization using the digoxigenin-alkaline phosphatase (DIG-AP) approach (Supplementary Table S2). Isolated brains were fixed in 4% PFA for 18 h (overnight) at 4 °C and then embedded in paraffin (Sigma-Aldrich, St. Louis, MO, USA) according to Boltana et al. [8]. Adjacent dorsal sections (7 µm) were washed twice with xylene, followed by decreasing concentrations of ethanol and finally washed twice in Tris-buffered saline (TBS) containing 0.05% Tween-20 (TBST). Sections were post-fixed with 4% PFA for 20 min and then treated with proteinase K (0.5 µg/mL; Sigma-Aldrich, St. Louis, MO, USA) in TBS with CaCl_2_ (2 mM) for 15 min at 37 °C and fixed again for 5 min. Endogenous phosphatase activity was blocked by acetylation treatment (0.25% acetic anhydride in 0.1 M triethanolamine, pH 8.0; Sigma-Aldrich, St. Louis, MO, USA) for 10 min with gentle agitation. Samples were pre-hybridized in hybridization buffer for 1 h in a humid chamber containing 2X SSC/50% formamide. Hybridization was performed overnight at 60 °C using 500–1000 ng/mL of the sense and antisense probes. To synthesize DIG-AP riboprobes, the cloned PCR product was generated with specific primers for each mRNA containing the T3/T7 RNA polymerase promoter sequence (Supplementary Table S1). Thin sections were hybridized with antisense and sense (negative control) riboprobes. After hybridization, sections were washed in 2× SSC and incubated in 2× SSC/50% formamide for 30 min at 60 °C, followed by washes in 2× SSC for 15 min at 60 °C, twice in 0.1× SSC at 60 °C, and finally in TBST. Staining was conducted by incubating the sections in Nitroblue tetrazolium/5-bromo-4-chloro-3-indolylphosphate (Roche, Basel, Switzerland) in the dark. After being air-dried, the slides were mounted using Faramount mounting medium (Dako). Pictures were taken using an Olympus AX70 microscope, and images were processed in ImageJ software (see details in the Supplementary Material).

### 4.6. ELISA Measurement of Plasma Cytokines

Blood plasma was obtained from ten previously sampled fish (see details Section 4.4) and stored at –80 °C until used. To determine the concentration of Il6, Tnfα1, Il1β, and annexin-1A in plasma samples, indirect ELISA was performed according to Morales-Lange et al. [69]. Briefly, each plasma sample was diluted once in carbonate buffer (60 mM NaHCO_3_, pH 9.6), loaded (in duplicated for each marker) at 35 ng/µL (100 µL) in a Maxisorp plate (Nunc, Thermo Fisher Scientific, Waltham, MA, USA), and incubated overnight at 4 °C. Afterwards, each well was blocked with 1% bovine serum albumin (BSA) for 2 h at 37 °C, and serum samples were incubated for 90 min at 37 °C with the primary antibody anti-synthetic epitope of Tnfα1 (diluted 1:500), Il6 (diluted 1:500), Il1β (diluted 1:500), and annexin-1A (1:500) in BSA [70,71,72]. Later, samples were incubated 60 min at 37 °C in the secondary antibody-HRP (diluted 1:7000, Thermo Fisher Scientific, Waltham, MA, USA). Finally, 100 µL of the chromogen substrate 3,3′,5,5′-tetramethylbenzidine (TMB) single solution (Invitrogen, CA, USA) was added per well and incubated for 30 min at room temperature. After adding 50 µL of 1 N sulfuric acid to stop the chromogenic reaction, plates were read at 450 nm on a VERSAmax microplate reader.

Primary antibodies against cytokines were produced according to Bethke et al. [70]. Recombinant proteins (TNFα1 and Il1β) and synthetic epitope peptides (Il6) were used for immunization in CF-1 mice (TNFα1 and Il6) [71] and New Zealand rabbit (Il1β [72] and recombinant annexin-1A [71]). For validation, antibody efficiency was determined by the calibration curve of the antibody against the recombinant proteins and the synthetic peptide used for immunization through indirect ELISA [73]. Its specificity was verified by Western blot, as described before in [72] (Supplementary Figures S1–S3). Measurement of plasma prostaglandin E2 (PgE2) levels was conducted using a commercial monoclonal enzyme immunoassay (EIA) according to the manufacturer’s instructions (Cayman, MI, USA). This assay has a range from 8.6 to 2000 pg/mL and sensitivity (80% B/B0) of approximately 30 pg/mL. Prior to PgE2 levels determination, plasma samples were diluted five times in EIA assay buffer.

### 4.7. Norepinephrine Detection by High-Performance Liquid Chromatography with Fluorescence Detection (HPLC-FL)

Norepinephrine (Ne) was detected from the brain and spleen, as described in Thomas et al. [74], with modifications. Briefly, samples were homogenized with 500 µL of 0.4 M perchloric acid per 0.4 g tissue using a Douncer homogenizer on ice. After 20 strokes, the homogenate was centrifuged at 13,523× *g* for 15 min at 4 °C, and the supernatant was filtered through 0.22 µm nylon filter and then injected in a HPLC-FL system. For chromatography separation, a high-pressure liquid chromatograph was used (Merck Hitachi). It consisted of an L-6200 pump, D-6000 interphase, an AS-4000 autosampler, and a F-1080 fluorescent detector. The chromatography separation was achieved on a LiChroCart Purospher STAR RP-18 chromatography column (250 × 4.6 mm, 5 µm size particle). The column temperature was maintained at room temperature (21 °C) during the experiment, with a flow rate of 1.0 mL/min. The mobile phase was composed of heptane-1 sulfonic acid sodium (11 mM), o-phosphoric acid (16.5 mM), and acetonitrile (11.5%), and the pH was adjusted to 7.1 with ammonium hydroxide (25%). The fluorescence intensity was monitored at excitation and emission wave at 279 and 320 nm, respectively. The volume injection of samples was 20 µL, and the total time of analysis per sample was 15 min. Peaks were identified by comparing their retention time in the sample solution (8.2 min) with the standard solution. Ne standard (Sigma-Aldrich, St. Louis, MO, USA) was used to make the stock solution (1.27 × 10^−4^ mM), which was prepared in nanopore water and stored in the dark at 4 °C. Working solutions were diluted with nanopore water and used immediately.

### 4.8. Choline/Acetylcholine Assay

Choline/ACh levels in the brain and spleen were measured using the ACh kit (Abcam, Cambridge, UK), according to manufacturer’s instructions. Briefly, organs were washed with cold 1X phosphate buffered saline (PBS), resuspended in 500 µL of choline assay buffer, homogenized with Douncer homogenizer on ice, and centrifuged (5283× *g* for 5 min at 4 °C), and the supernatants were collected and kept on ice. To determine total choline, 50 µL of sample was mixed with 50 µL of reaction solution containing choline assay buffer, choline probe, enzyme, and AChE. The reaction was incubated for 30 min in the dark and at room temperature. Absorbance was measured at 570 nm on Multiskan Go (ThermoScientific, Waltham, MA, USA). ACh levels were determined using the following equation:

*Acetylcholine* = *Total choline - Free choline*

### 4.9. Cholinesterase Enzymatic Activity Assay

The AChE activity in the brain and spleen was measured using Ellman’s method [75]. Briefly, acetylcholine was used as substrate and hydrolysed by AChE, generating two products: acetic acid and thiocholine. Thiol continuous reaction with 5,5-dithio-bis-2-nitrobenzoate (DTNB) ion was used to produce the yellow anion 5-thio-2-nitrobenzoic (TNB) acid. The brain and spleen were homogenized with 1 mL of PBS 1X (pH 8) using the Tissue-Lysser II (Qiagen) at 25 Hz for 5 min and centrifuged at 5283× *g* for 15 min at 4 °C. The supernatant was then collected and immediately used. To determine AChE activity, 100 µL of samples were mixed with 25 µL of DTNB and incubated for 3 min at room temperature. Then, 5 µL of ACh iodide solution was added to the reaction mix. The change in absorbance per minute was measured at 412 nm using Multiskan Go (ThermoScientific, Waltham, MA, USA) for 5 min. The rate of AChE activity was estimated and expressed as μmol/min/mg total protein using Lowry’s method. Enzymatic activity of AChE (U/L) was calculated according to Jiménez-Díaz and Martínez-Monge [76], with modifications. The molar absorptivity of TNB was 14, 15 nM^−1^ cm^−1^ at 412 nm [77]

$$Acetylcholinesterase\left(U/L\right)=\Delta A/min\times \frac{Total\;volume\;}{sample\;volume\times molar\;absorptivity\;TNB}$$

### 4.10. Lipid Content

Lipids were extracted from fish liver (3 fish per time and treatment), as described by Bligh and Dyer [78]. Subsequently, transesterification was performed by adding 2 mL of borontrifluoride (BF3)/methanol (12%) and then incubating at 100 °C for 30 min. After cooling the samples, 1 mL of isooctane was added, followed by stirring. To separate the phases, 2 mL of saturated NaCl was added. The upper phase was transferred into 2 mL amber vials and dried under a stream of nitrogen. Then, it was resuspended in 100 µL of hexane and analysed on a gas chromatograph, where a mixture of 36 fatty acid methyl esters (FAME) (Restek, Food Industry FAME MIX) was used as standard. The temperature range to allow chromatography was 100 °C to 240 °C, maintaining the final temperature for 20 min. The injection volume was 2 µL per sample, and the carrier gas was nitrogen at 100 kPa. The injector temperature was 225 °C, and the FID detector temperature was adjusted to 250 °C. The data were obtained using the Autochro Data Module interface and Autochro 3000 software (Young Lin Instrument). The FAME profiles of the samples were identified by comparing the retention times and the area of the FAME peaks (mV) with the standard.

### 4.11. High-Throughput Transcriptome Profiling: Library Construction, Illumina Sequencing, and Data Analysis

Total isolated RNA from the spleen of five randomly selected individuals belonging to each experimental condition (control, no-fever, fever) was pooled (*n* = 5 individuals per pool and three replicates by condition, GEO-NCBI repository GSE 141554) at 24 hpi. RNA extraction was individually isolated, and genomic DNA was removed using Ribo-PureTM Kit (Ambion^®^, Austin, TX, USA) and DNase I treatment (Fermentas, MA, USA), respectively, according to the manufacturer’s instructions. RNA integrity number (RIN) was evaluated through the 2200 TapeStation (Agilent Technologies, Santa Clara, CA, USA) using the R6K ScreenTape (Agilent Technologies, Santa Clara, CA, USA). Samples with high RIN values (RIN ≥ 8; 260/280 ratio ≥ 1.8) were used for library construction. Total RNA was quantified with Qubit^®^ 2.0 Fluorometer (Invitrogen, CA, USA) (*n* = 3 pools per treatment). Fifteen barcoded libraries were generated using the KAPA Stranded mRNA-Seq Kit (KapaBiosystems, Wilmington, MA, USA) according to the manufacturer’s instruction, which where validated based on length distribution as estimated with the 2200 TapeStation (Agilent Technologies, Santa Clara, CA, USA) using D1K screen tape and reagents (Agilent Technologies, Santa Clara, CA, USA). Three biological replicates (*n* = 15 fish total per condition) were used for sequencing, which was performed using libraries with mean length peaks above 250 bp and quantified by qPCR using the Library Quantification Kit Illumina/Universal (KapaBiosystems, MA, USA) according to the manufacturer’s instructions. RNA-Sequencing (RNA-Seq) was performed with the Miseq (Illumina) platform using a run of 2 × 250 paired-end reads at the Universidad de Concepción, Chile. Raw reads for both conditions were mapped on the *Salmo salar* genome annotation (reference Atlantic salmon genome and gene model annotation files were downloaded directly from the National Center for Biotechnology Information (NCBI) Atlantic salmon database, and the accession code was GCA_000233375.4, [79]) using Tophat2 software [80]. The mapped reads were assembled into transcripts using the annotated transcriptome of *Salmo salar* as reference through implementation in the Cufflinks2 V2.2.0 package [81]. To consolidate transcriptome assembly and quantify its estimated expression as normalized fragments per kilobase million (FPKM) values, we used the Cuffmerge and Cuffquant packages (Cufflinks2 V2.2.0), respectively. Differential gene expression (DGE analysis) was performed using the Cufflinks2 V2.2.0 package [81], considering a gene differentially expressed with a false detection rate (FDR) < 0.05. 

### 4.12. Gene Ontology (GO) Enrichment and Interactome Analysis

For gene ontology (GO) mapping, the GO terms and DEGs in the spleen samples were extracted using DAVID Bioinformatics Resources (GO-DAVID analysis; http://david.abcc.ncifcrf.gov/tools.jsp) [35,82]. Then, to examine the enriched functional-related gene groups, we used the Biological Network Gene Ontology (ClueGO, version 2.0) plugin [36]. It allowed statistical evaluation of groups of gene products with respect to the current annotations available at the Gene Ontology Consortium (http://www.geneontology.org). To identify interacting networks, we used the Cytoscape 3.5.1 software (http://www.systemsbiology.org). Enrichment of each GO term was evaluated using Fisher’s exact test and corrected for multiple testing with FDR < 0.05 [83]. We applied a Bonferroni correction to account for the multiple tests performed. Each gene set comprised at least 4 transcripts that shared the same GO biological process or annotation term. Topological analysis of individual and combined networks was performed using Network Analyzer. The jActiveModules 2.2 software was used to analyse network features [84,85].

### 4.13. Pharmacological and Functional Studies

The pharmacological study was conducted in cultured macrophages isolated from the spleen and treated with the selective α7 nicotinic ACh receptor agonist (ACh, Sigma-Aldrich, CAS # 60-31-1) and the antagonist α-bungarotoxin (α-BTX, ab120542; Abcam, Cambridge, MA, USA). The structurally unrelated competitive antagonists α-BTG (peptide) exclusively bind to the Chrna7 at nanomolar concentrations [37]. Briefly, fish macrophages from spleens were cultured as described previously [86] using Dulbecco’s modified Eagle medium (DMEM) 4.5g/l glucose supplemented with 10% (*v*/*v*) heat-inactivated foetal bovine serum (FBS) and Primocin (Invivogen), and incubated at 15 °C and 5% CO_2_. Spleen cells were seeded in 6-well plates at 80% confluence. The cells were challenged with IPNV, and then 1.5 µg/mL of α-BTX and ACh was added and left for 1 h at room temperature (see details in [23]). The inflammation-inducing treatments were performed as follows: control, α-BTX, ACh, α-BTX-IPNV, and ACh-IPNV. Control cells were treated with saline solution (10 µL of 1X PBS). Cell culture supernatants (triplicates) from 3 fish were recovered, centrifuged, and stored at −80 °C until use. Il10 and Il1β levels were measured using a polyclonal EIA assay, as described by [69]. The methodology, antibodies, and reagents used to determine Il1β levels are described in Section 4.6. After blocking in 1% BSA for 2 h, cells were incubated for 90 min at 37 °C in the primary antibody anti-synthetic epitope of Il1β (diluted 1:500) in blocking solution. Cells were then washed and incubated for 60 min at 37 °C in the secondary antibody-HRP (diluted 1:7000, Thermo Fisher Scientific, Waltham, MA, USA). Measurement of Il10 levels was performed using a commercial primary polyclonal antibody anti-Zebrafish Il10 (diluted 1:500, Kingfisher Biotech, Inc., St. Paul, MN, USA) and an ELISA assay kit according to the manufacturer’s instructions (Kingfisher Biotech, Inc., KP1267Z-100, St. Paul, MN, USA). Before Il10 levels determination, supernatants were diluted four times in the ELISA assay buffer.

### 4.14. Flow Cytometry Analysis

Leukocytes were isolated from salmon spleen and processed for flow cytometry analysis performed in each condition: control, fever, no-fever. These cells were isolated by disaggregating tissue through Falcon^®^ cell strainers (100 µm) with DMEM media plus 10% FBS and 1% Glutamax and pelleted by centrifugation at 135× *g* for 10 min. To initially identify salmon CD4-bearing cells, we stained spleen leukocytes with salmon anti-CD4-1 (5 mg/mL) primary monoclonal antibody (mAb). Stained cells were detected with Brilliant Violet 421 (565014, BD Bioscience, Franklin Lakes, NJ, USA). To elucidate whether spleen cells expressed CD4-1+ and/or CD83+, we stained spleen leukocytes with biotinylated salmon anti-CD4-1 (5 mg/mL) and salmon anti-CD83 (1 mg/mL) primary mAbs. Stained cells were detected with Brilliant Violet 421 Streptavidin (1 mg/mL; 565014 BD Bioscience, Franklin Lakes, NJ, USA) and mouse IgG-Alexa Fluor 488 (1 mg/mL; ab150113, Abcam, Cambridge, UK) mAbs. Cell counting was performed as described in Maisey et al. [56] and Nombela et al. [87], with modifications. Briefly, spleen cells were re-suspended and blocked with 1X PBS plus 2% FBS immunofluorescence (IF) media. Cells were then incubated for 1 h at 4 °C with the primary antibodies anti-CD4-1 and anti-CD83 [87,88]. Cells were incubated for 1 h at 4 °C in IF media containing secondary antibodies BV421 Goat Anti-Rabbit IgG, Clone Polyclonal (565014, BD Bioscience, Franklin Lakes, NJ, USA), and Alexa Fluor^®^ 488 Goat Anti-Mouse IgG H&L (ab150113, Abcam, Cambridge, UK). Finally, the leukocytes were washed, centrifuged, and suspended once again in 200 µL of IF media before analysis. For autofluorescence measurement, cells were suspended with IF solution without antibody. As negative controls, cells were treated with the corresponding conjugated secondary antibody only. For fluorescence-activated cell sorting (FACS), a BD LSRFortessa™ X-20v cell analyser was used to estimate the cell count, and at least 10,000 events were recorded for each sample. Recorded events were analysed using the FlowJo software.

### 4.15. Statistical Analysis

The presented data are expressed as mean ± standard deviation (SD). Sigma Plot (version13.0) and GraphPad Prism 7 software were used for statistical analysis and graphing, respectively. For locomotor activity analysis, one-way ANOVA (ordinary), two-way ANOVA on ranks (Kruskal–Wallis), and Mann–Whitney U tests were used to compare the treatments and were corrected by applying multiple comparison tests with Dunnett’s and Tukey’s adjustments. Complementarily, to inspect whether the fish had preferences for any chamber or temperature, a Wald test was applied. A behavioural fever analysis was performed using generalized Poisson regression models, as described in Section 4.3. For RNA-seq analysis, multiple comparisons between groups developing fever were conducted when the overall *p*-value was significant, using the Bonferroni method for adjusting the level of significance. Predictions of fish numbers from final models were calculated and plotted to evaluate interactions. All statistical analyses were performed using SigmaPlot version 14 (SigmaPlot, Stata Statistical Software). Non-parametric statistics were also used when the normality test failed. Means and significance levels for the groups were compared with controls. The significance indicator or a letter above each graph represents a significant difference relative to control. A *p* < 0.05 was considered statistically significant.

## 5. Conclusions

This study generated a molecular map of the inflammatory reflex in fish, triggered during behavioural fever. Our findings show, for the first time, that fever in mobile ectotherms leads to a neuro-immune interaction, which might modulate the systemic inflammatory response under pathogen infection. Our work points to a conserved neural–immune link contributing to a better understanding of the pathogen-mediated inflammatory disease and immune disorders responses during metazoan evolution. The present data reinforce the neural–immune synergy in thermal stimulation. Such a synergistic effect has implications for considering the unpredictable outputs of fish rearing at constant temperatures in aquaculture or laboratory settings. Additionally, it is well known how animals sense temperature, but how heat-induced stimuli influence organismal behaviour is poorly understood. Our molecular characterization opens the door for further heat-related gene functions discovery in a genetic model. It also serves as a tool for elucidating how environmental thermal sensations lead to specific behaviours at the single-molecule level. Importantly, our study also provides a hypothetical model that is expected to aid in understanding the impact of global warming on thermoregulatory behaviour in ectotherms.

## Data Availability

The RNA-Seq data supporting the conclusions of this article are available in the Gene Expression Omnibus (GEO): GSE141554.

## Supplementary Materials

The following are available online at https://www.mdpi.com/article/10.3390/ijms22168860/s1, Figure S1: Behavioural fever in IPNV-challenged *Salmo salar*, Figure S2: Tnfα antibody development and validation, Figure S3: Il1β antibody development and validation, Figure S4: Il6 antibody development and validation, Figure S5: Spatially-defined expression of the *il1* receptor (*il1r*) transcript in the brain of virus challenged *Salmo salar*, Table S1: mRNA primer sequences used for absolute RT-qPCR analysis title

## Abbreviations

Chrna7	Cholinergic receptor nicotinic alpha 7 subunit;
Adrb2	Beta-2 adrenergic receptor
Ach	Acetylcholine
Tnfα	Tumour necrosis factor alpha
Il1β	Interleukin-1 beta
Il6	Interleukin-6
Il10	Interleukin-10
Tgfβ	Transforming growth factor β
PgE2	Prostaglandin E2
qPCR	Quantitative polymerase chain reaction
MS-222	Tricaine methane sulphonate
α-BTX	α-bungarotoxin
PBS	Phosphate-buffered saline
