# Supplementary material for: Behavioural Fever Promotes an Inflammatory Reflex Circuit in Ectotherms
Source: Int J Mol Sci. 2021 Aug 17;22(16):8860. doi: 10.3390/ijms22168860 (PMC8396262; doi:10.3390/ijms22168860)
Supplement: Supplementary file 1 [file ijms-22-08860-s001.zip › Sanhueza et al, IJMS. Supplementary File.pdf]

Supplementary Material

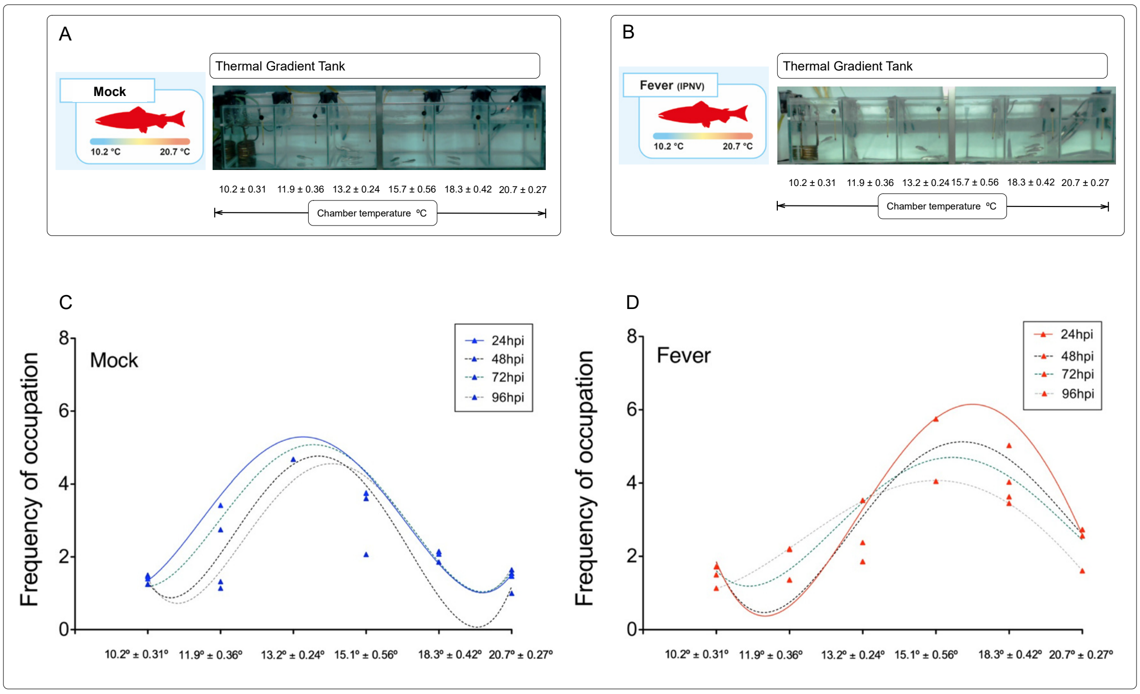

Supplementary Figure S1 - Behavioural fever in IPNV-challenged *Salmo salar*. (A) Schematic (left) and image (right) of the fish exposed to 100 ml virus-free cell culture supernatant (mock-infected individuals) in the fever-tank. **(B)** Schematic (left) and image (right) of the fish challenged with IPNV (virus-infected individuals) in the fever-tank. **(C)** The blue triangles represent the frequency of occupation in each chamber of non-challenged individual fish (mock-infected individuals) in each time post treatment (24, 48, 72, and 96 hours post infection, hpi). **(D)** The red triangles represent the frequency of occupation in each chamber of fish challenged with IPNV under thermal gradient tank (n = 10 individuals per replicate, mean ± standard deviation (SD)).

Antibodies development and validation for cytokine detection levels

To investigate the levels of immunoreactive cytokines detected by ELISA (see Material and methods section), we produced a specific set of antibodies against Tnfα, Il1β and Il6. The development and validation strategies of the antibodies used here are similar to those reported for other research groups (Bethke *et al*, 2012; Rojas *et al.*, 2012; Schmitt *et al.*, 2015). We used two immunization strategies: a) a recombinant molecule (Supp. Fig. 2B,3B) and b) a synthetic epitope peptide as the antigen (Supp. Fig. 4B). For antibody validation we evaluated the affinity and specificity of the antibody/antigen complex by ELISA and Western blot, respectively. The Tnfα and Il1β antibodies specifically detect their salmonid versions (Supp. Fig. 2A,B and 3A,B). The Il6 antibody specifically recognizes an

18 amino acids length epitope of the protein (Supp. Fig. 4A), and its monomeric and dimeric forms (Supp. Fig. 4B).

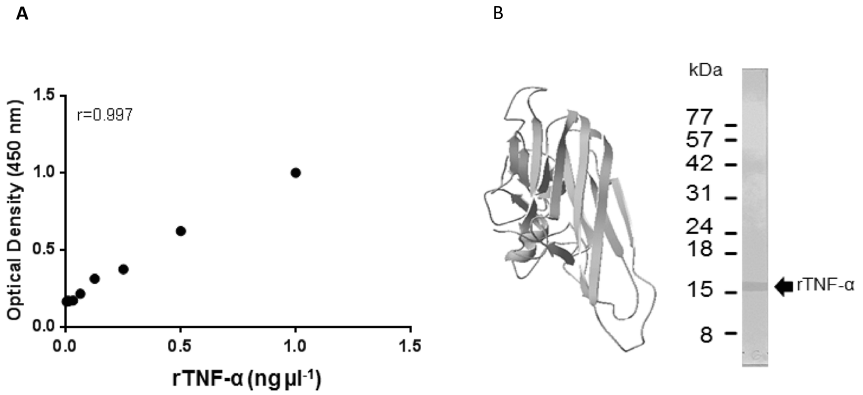

Supplementary Figure S2 - Tnfα antibody development and validation. (A) The affinity and specificity of the antibody were assessed by ELISA (r-value= ~1 indicates high affinity of the antibody-antigen interaction). **(B)** Left: the antibody against the salmonid cytokine Tnfα was developed using its recombinant version (rTnfα**)** as the antigen. Molecular modeling of the rTnfα shows its tridimensional structure. Right: antibody validation by western blot using challenged (infected) fish samples. Black arrow indicates the immunoreactive band. ng ul^{-1}: nanograms per microlit er; nm: nanometers; kDa: kilodaltons.

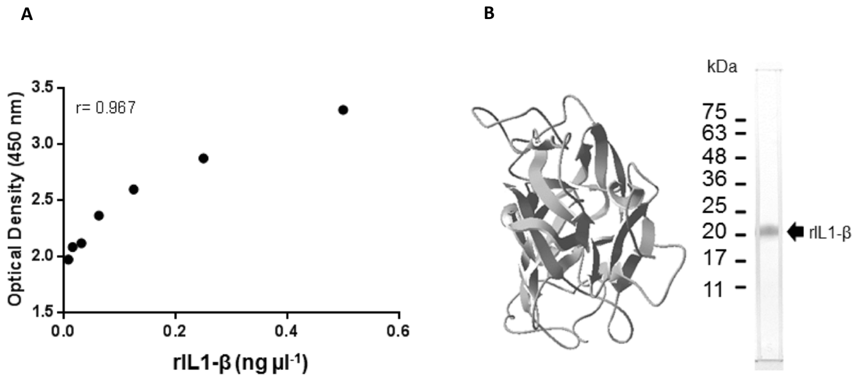

Supplementary Figure S3 - Il1β antibody development and validation. (A) The affinity and specificity of the antibody were assessed by ELISA (r-value= ~1 indicates high affinity of the antibody-antigen interaction). **(B)** Left: the antibody against the salmonid cytokine Il1β was developed using its recombinant version (rIl1β) as the antigen. Molecular modeling of the rIl1β shows its tridimensional structure. Right: antibody validation by western blot using challenged (infected) fish samples. Black arrow indicates the immunoreactive band. ng ul^{-1}: nanograms per microliter; nm: nanometers; kDa: kilodaltons.

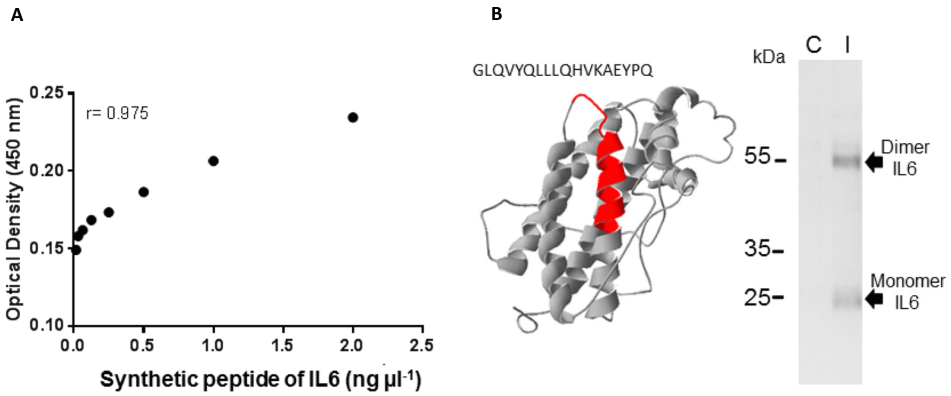

Supplementary Figure S4 - Il6 antibody development and validation. (A) The affinity and specificity of the antibody were assessed by ELISA (r-value= ~1 indicates high affinity of the antibody-antigen interaction). **(B)** Left: the antibody against the salmonid Il6 was developed using a synthetic epitope peptide (in red). Molecular modeling of the Il6 cytokine shows that it consists in a highly conserved 18 amino acids length region of the protein and its tridimensional structure. Right: antibody validation by western blot using control (C, uninfected) and challenged (I, infected) fish samples. Black arrows indicate the immunoreactive bands in the challenged fish samples corresponding to the IL-6 monomeric monomer (lower band) and dimeric (upper band) forms. ng ul^{-1}: nanograms per microliter; nm: nanometers; kDa: kilodaltons.

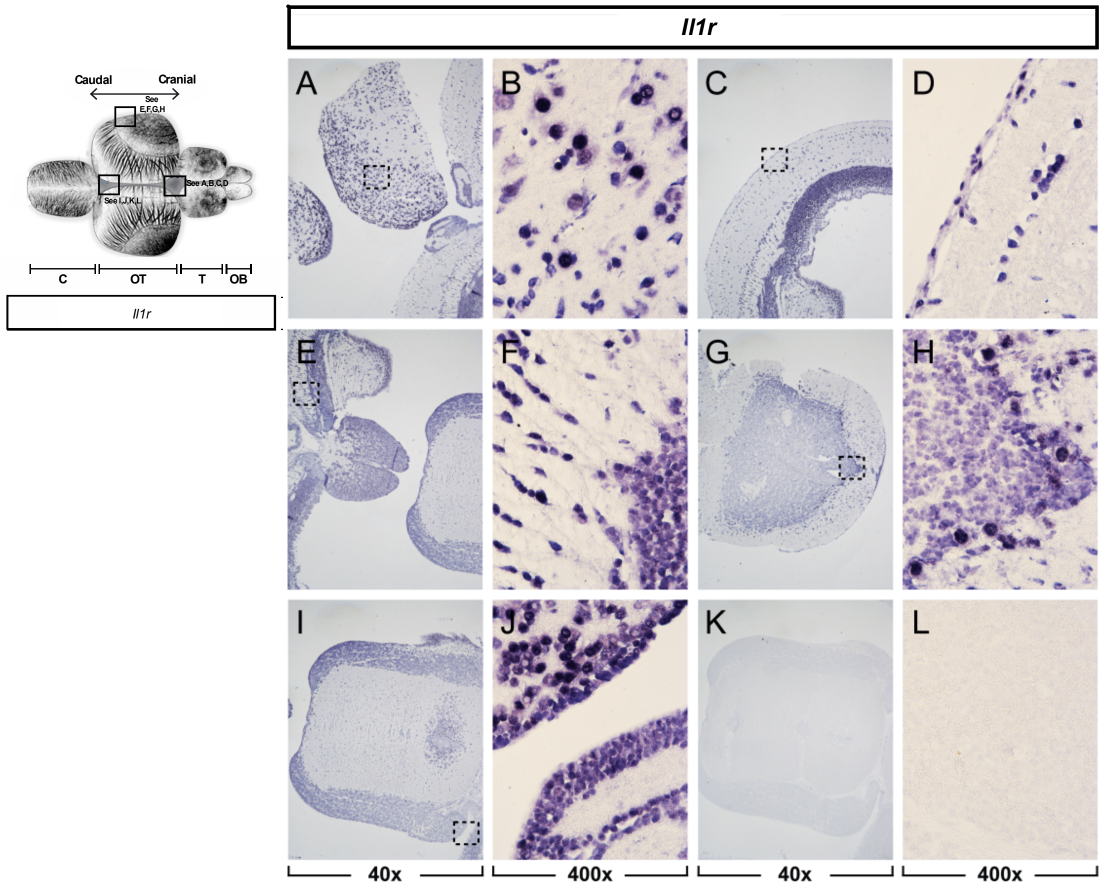

Supplementary Figure S4 – Spatially-defined expression of the *il1* receptor (*il1r*) transcript in the brain of virus challenged *Salmo salar*. The left panel shows the different brain regions in which the expression of the *il1r* mRNA was examined (black squares). The right panels show brain images for the distribution of the transcript detected by *in situ* hybridization. **(A-J)** Expression and spatial localization of the *il1r* transcripts in a sectioned brain of an infected fish. Insets (dashed line squares) are showing the cell-specific distribution of the mRNA in the telencephalon and optic lobes. **(K, L)** Sectioned brain images showing negative control (sense riboprobe) staining. C: Cerebellum; OT: Optic lobes; T: Telencephalon; OB: olfactory bulb. Scale bar= 25 µm.

Supplementary Tables

Supplementary Table S1- mRNA primer sequences used for absolute RT-qPCR analysis.

**Gen**	**Name**	**Primer 5'-3'**	Amplicon (pb)	**E (%)**	**Tm (°C)**	**GenBank**
il10	*interleukin 10*	F: ATGAGGCTAATGACGAGCTGGAGA<br>R: GGTGTAGAATGCCTTCGTCCAACA	54	102	60	EF165028
tgfβ	*transforming growth factor Beta 1*	F: AACCGGGCTAGCATTAACCT<br>R: TTGACCTCTGTCCAGCACAC	173	99	56	X99303.1
il1r	*interleukin-1 receptor-like protein* (*il1rl*)	F: ACCAGCAGATTGGCCTGTAC<br>R: TGCGGTTTCAGATCCCCTTT	250	114	60	NM_001123633.1
tnfr	*tumor necrosis factor receptor superfamily member 1A* (*tnr1a*)	F: TCTACTCGGAGGCTGATCCA<br>R: GGAGGTGGTCATTCTCAGCC	250	112	60	NM_001141773.1
tnfα	*tumor necrosis factor-Alpha*	F: AGGTTGGCTATGGAGGCTGT<br>R: TCTGCTTCAATGTATGGTGGG	250	108	62	NM_001123589.1
il1β	*interleukin 1 Beta*	F: CGTCACATTGCCAACCTCAT<br>R: ACTGTGATGTACTGCTGAAC	200	98	60	AY617117
adrb2	*beta2-adrenergic receptor*	TACGCAAGATTGACCGCAGT<br>CAGCGTGAAGATCCCCATGA	200	89	64	XM_014213101.1
chrna7	*α7 nicotinic receptor*	F: CTGGCTTCGCAGAGTATCTCC<br>R: GTTGGACATGTACCCGGAGA	176	90	60	XM_021560667.1
WB117	*infectious pancreatic necrosis virus, protein VP2*	F: GCGGTTCGACTTCATTCTACA<br>R: GAGCTTGTCACGGAGACCAC	100	116	60	U48225.1
tbx21	Interleukin 12	F: AGTGAAGGAGGATGGTTCTGAG<br>R: GGTGATGTCTGCGTTCTGATAG	112	99	62	HQ450583
il2	*interleukin 2*	F: GTTGCAGCATTGGCCTGTTCC<br>R: AGTCCTGTTGATGTGGTGTTGC	110	99	60	FN356744.1

ifnγ	*interferon gamma*	F: CTAAAGAAGGACAACCGCAG	159	107	60	AJ841811.1
		R: CACCGTTAGAGGGAGAAATG				
stat4	*signal transducer and activator of transcription 4*	F: AGCCTGGGACTTTCCTGCTG	119	97	62,5	BT059211.1
		R: TTTGGTGTACGGCTCCACCG				
		R: GAGCTTGTCACGGAGACCAC				

Supplementary Table S2-Primer sequences used for *in situ* hybridization

Target gene	Gene name	Primers	Primer sequence (5′→ 3′)
il1rl	interleukin-1 receptor-like protein	[a]5809 (Fw)	T3Rpps-ATGTATTCACATGGCTATTATGCCTATGGC
		[b]5810 (Rv)	T7Rpps-CAGTAAAAACCTTGGTTGAGACCATGCTGT

[a] Primers 5809, 5811, 5813, 5815, 5817 and 5821 contain the T3 RNA polymerase promoter sequence (underlined) at their 5′-ends (T3Rpps; 5′-GGGCGGGTGTTATTAACCCTCACTAAAGGG-3′).

[b] Primers 5810, 5812, 5814, 5816, 5818 and 5822 contain the T7 RNA polymerase promoter sequence (underlined) at their 5′-ends (T7Rpps; 5′-CCGGGGGGTGTAATACGACTCACTATAGGG-3′).

Fw, forward; Rv, reverse.
